# Chemopreventive Effect of Phytosomal Curcumin on Hepatitis B Virus-Related Hepatocellular Carcinoma in A Transgenic Mouse Model

**DOI:** 10.1038/s41598-019-46891-5

**Published:** 2019-07-17

**Authors:** Chiao-Fang Teng, Chun-Hui Yu, Hong-Yi Chang, Wen-Chuan Hsieh, Tzu-Hua Wu, Jia-Hui Lin, Han-Chieh Wu, Long-Bin Jeng, Ih-Jen Su

**Affiliations:** 1 Graduate Institute of Biomedical Sciences, China Medical University, Taichung, Taiwan; 2 Organ Transplantation Center, China Medical University Hospital, Taichung, Taiwan; 3 Department of Biotechnology, Southern Taiwan University of Science and Technology, Tainan, Taiwan; 4 National Institute of Infectious Diseases and Vaccinology, National Health Research Institutes, Tainan, Taiwan; 5 Department of Pathology, National Cheng Kung University Hospital, Tainan, Taiwan

**Keywords:** Cancer prevention, Hepatocellular carcinoma

## Abstract

Chronic hepatitis B virus (HBV) infection is a major risk factor for the development of hepatocellular carcinoma (HCC), a leading cause of cancer mortality worldwide. Hepatitis B X protein (HBx) and pre-S2 mutant have been proposed as the two most important HBV oncoproteins that play key roles in HCC pathogenesis. Curcumin is a botanical constituent displaying potent anti-inflammatory and anti-cancer properties without toxic side effects. Phytosomal formulation of curcumin has been shown to exhibit enhanced bioavailability, improved pharmacokinetics, and excellent efficacy against many human diseases. However, effectiveness of phytosomal curcumin for HCC treatment remains to be clarified. In this study, we evaluated chemopreventive effect of phytosomal curcumin on HBV-related HCC by using a transgenic mouse model specifically expressing both HBx and pre-S2 mutant in liver. Compared with unformulated curcumin, phytosomal curcumin exhibited significantly greater effects on suppression of HCC formation, improvement of liver histopathology, decrease of lipid accumulation and leukocyte infiltration, and reduction of total tumor volume in transgenic mice. Moreover, phytosomal curcumin exerted considerably stronger effects on activation of anti-inflammatory PPARγ as well as inhibition of pro-inflammatory NF-κB than unformulated curcumin. Furthermore, phytosomal curcumin showed a comparable effect on suppression of oncogenic mTOR activation to unformulated curcumin. Our data demonstrated that phytosomal curcumin has promise for HCC chemoprevention in patients with chronic HBV infection.

## Introduction

Hepatocellular carcinoma (HCC) is one of the most common and lethal cancers worldwide, killing more than 500,000 people every year^1–3^. Chronic hepatitis B virus (HBV) infection is a major risk factor for HCC development, accounting for over 50% of total cases worldwide^4,5^. Several mechanisms have been proposed to explain HBV-related hepatocarcinogenesis, including the insertional mutagenesis of HBV DNA, the inflammation and regeneration hyperplasia initiated by immune responses to HBV infection, and the oncogenic functions of HBV gene products^6,7^. Although anti-viral drugs are currently used to treat chronic HBV infection, the progression from chronic HBV infection to HCC remains a nightmare for the majority of chronic HBV carriers^8,9^. Even when the viral loads were significantly reduced or even made undetectable, the expression of HBV surface antigen remained persistently detectable, at least in part, due to the integration of HBV DNA into hepatocytes during the course of chronic HBV infection^10–12^. Moreover, the therapeutic efficacy of HCC is frequently impeded by side effects during long-term treatment^13,14^. Therefore, identification of chemopreventive agents with acceptable side effects remains a primary objective in the prevention of HCC in patients with chronic HBV infection.

Two HBV viral proteins, the hepatitis B X protein (HBx) and pre-S2 mutant large hepatitis B surface antigen (HBsAg), have been well demonstrated as oncoproteins that exhibit either direct or indirect oncogenic effects in the liver of chronic HBV carriers, contributing to the progression of HCC^15,16^. HBx mediates the activation of multiple signal pathways in hepatocytes to regulate a variety of cellular functions, including the cell cycle, proliferation, apoptosis, metabolism, and DNA repair^17,18^. Besides HBx, our previous studies have shown that pre-S2 mutant is accumulated in the endoplasmic reticulum (ER) of type II ground glass hepatocytes (GGHs) and induces multiple ER stress-dependent and -independent signal pathways, leading to growth advantage and genomic instability^19–21^. Most recently, we have also shown that intrahepatic HBV large surface antigen can impair hepatocyte cytokinesis, resulting in hyperploidy, contributing to the development of HCC in chronic HBV carriers^22^. Moreover, we have observed the co-expression of HBx and pre-S2 mutant in type II GGHs in patients with chronic HBV infection^23^. Hepatocytes co-expressing HBx and pre-S2 mutant exhibit enhanced activation of the oncogenic mammalian target of rapamycin (mTOR) signal pathways induced by either HBx or pre-S2 mutant alone^23^. Transgenic mice harboring both HBx and pre-S2 mutant display consistently activated mTOR signal throughout the liver tumorigenesis and have significantly higher frequency and shorter time to develop HCC than mice carrying either viral protein alone^23^. Therefore, type II GGHs co-expressing HBx and pre-S2 mutant represent an important precursor lesion for HCC development in chronic HBV infection. Transgenic mice harboring the double HBV oncoproteins can be used as an ideal model to develop preventive and therapeutic drugs for HBV-related hepatocarcinogenesis.

Natural products have been considered as promising cancer chemopreventive agents owing to advantages such as multitarget properties, easy availability, low toxicity, and reduced production cost^24^. Among them, curcumin, a polyphenolic compound derived from the plant turmeric, is one of the best investigated botanical constituents and has a long history of use in the treatment of chronic diseases due to its anti-inflammatory effects^25,26^. Curcumin has been shown to exert its anti-inflammatory effects through activating peroxisome proliferator-activated receptor γ (PPARγ) that results in inhibition of pro-inflammatory nuclear factor-κB (NF-κB) activation^27,28^ as well as anti-cancer effects through blocking the oncogenic mTOR signal activation^29^. Several lines of evidence have shown that curcumin has both chemopreventive and therapeutic effects on HCC in human hepatoma cells and in model animals through multiple mechanisms, including inhibition of cell proliferation, migration, and invasion, as well as induction of apoptosis^30–35^. However, there have been no published reports regarding its effects on HBV-related HCC. Moreover, its effectiveness and clinical application have been limited due to poor bioavailability in human body^36^. As a result, different formulation strategies such as liposomes, solid dispersion, complex, emulsion, micelles, nanogels, and microspheres have been employed to improve the bioavailability of curcumin^37^. Most recently, phytosomes emerge as promising biocompatible carriers of natural drugs with a better stability than many other formulations^38^. Remarkably, phytosomal formulation of curcumin has been shown to display improved curcumin bioavailability and pharmacokinetics and exhibit excellent efficacy against several human diseases including cancer, diabetes, and inflammatory diseases^39–41^. Nevertheless, the effectiveness of phytosomal curcumin in the treatment of HCC remains to be clarified.

Therefore, in this study, we evaluated the chemopreventive effect of phytosomal curcumin on HBV-related HCC development by using the transgenic mouse model expressing both HBx and pre-S2 mutant. The inhibitory effect of phytosomal curcumin on the activation of pro-inflammatory and oncogenic signals was also determined and compared with the unformulated curcumin.

## Results

### Curcumin administration in transgenic mice expressing both HBx and pre-S2 mutant

**Figure 1:**
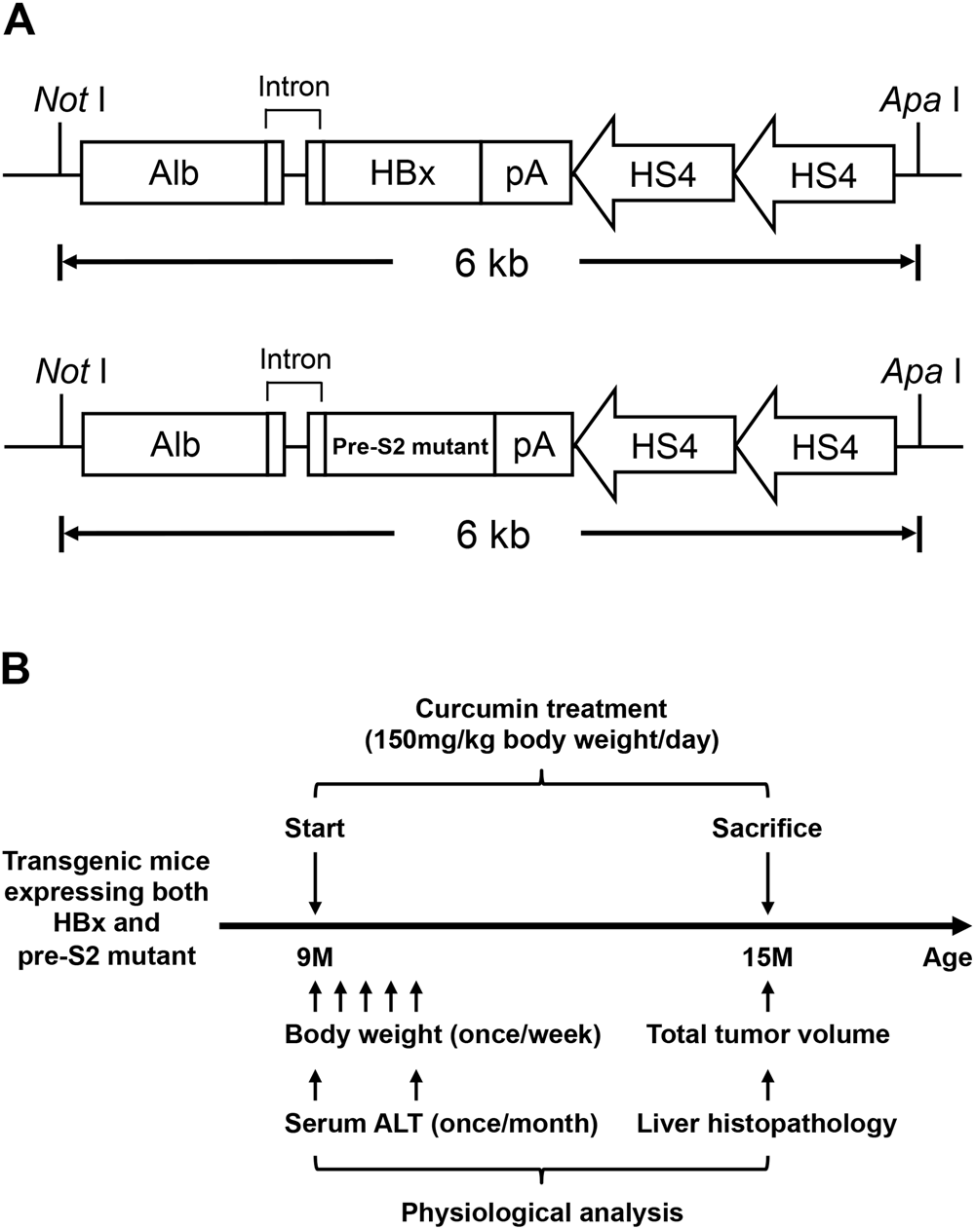
Establishment and curcumin treatment of the transgenic mouse model expressing both HBx and pre-S2 mutant. (**A**) Schematic diagram of the albumin-HBx and -pre-S2 mutant transgenic constructs. The HBx and pre-S2 mutant transgenes were driven by the liver-specific albumin promoter. The *NotI* and *ApaI* restriction enzyme sites were used to excise the 6-kb DNA insert for pronucleus microinjection. (**B**) Schematic diagram of curcumin treatment. The transgenic mice were treated with either normal diets or the diets containing unformulated curcumin or phytosomal curcumin (150 mg curcuminoids/kg body weight/day), beginning at 9 months of age, for 6 months, and were then sacrificed. The body weight and serum ALT of mice were measured immediately before treatment and routinely after treatment until sacrifice. At sacrifice, the total tumor volume of mice was measured and the histopathology of mice liver was examined.

The transgenic mice expressing both HBx and pre-S2 mutant in liver were established by Professor Ting-Fen Tsai’s laboratory as described^23,42,43^. Briefly, the transgenic mice were generated in the C57BL/6 background and the HBx and pre-S2 mutant transgenes were driven by the liver-specific albumin promoter (Fig. 1A). Considering that transgenic mice harboring both HBx and pre-S2 mutant revealed obvious liver pathology (such as hepatic inflammation and steatosis) around the age of 9 months and developed HCC at an average age of 15 months^23,42,43^, the mice were treated with the normal diets, unformulated or phytosomal curcumin diets, beginning at 9 months of age, for 6 consecutive months (until 15 months of age), and were then sacrificed for analysis (Fig. 1B).

### Phytosomal curcumin maintained normal body weight and serum alanine aminotransferase (ALT) level in transgenic mice expressing both HBx and pre-S2 mutant

**Figure 2:**
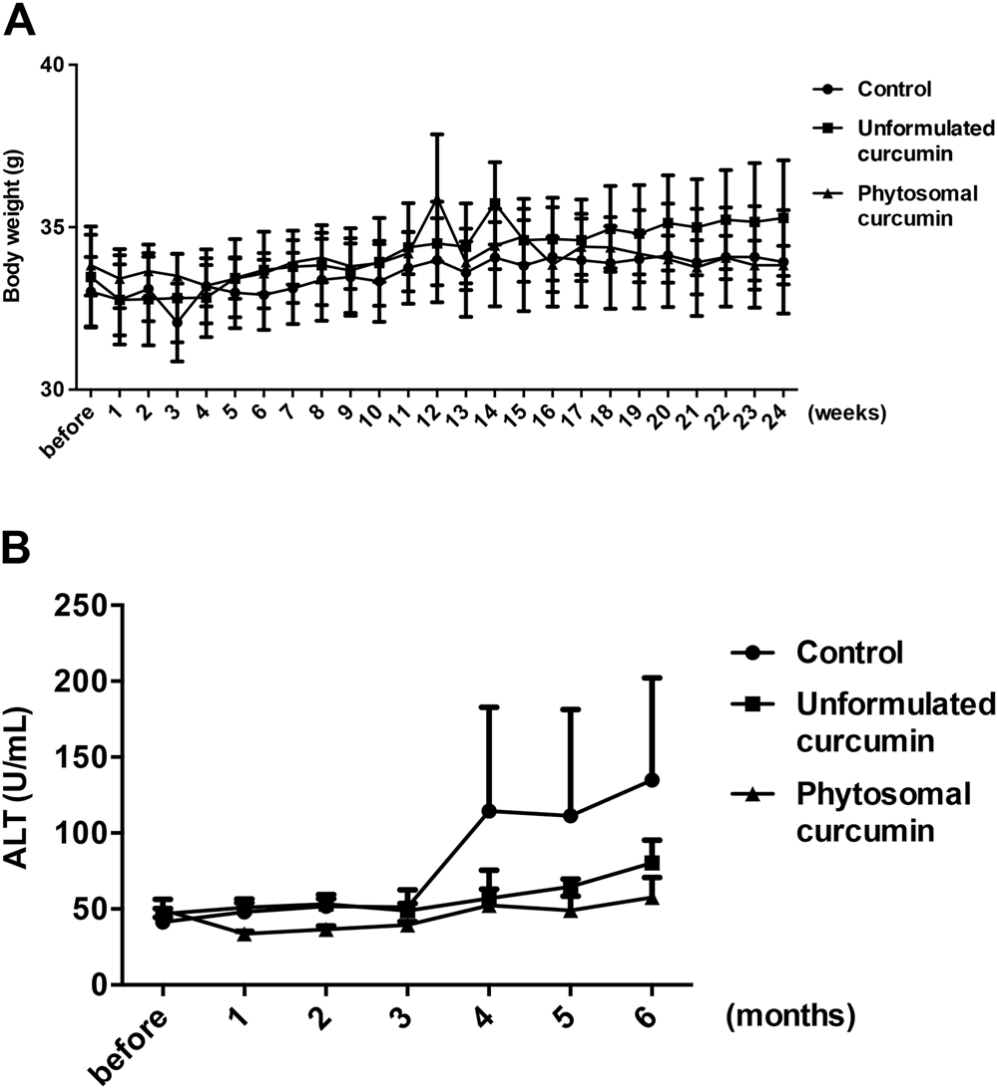
Phytosomal curcumin did not affect body weight and lowered serum ALT level in transgenic mice expressing both HBx and pre-S2 mutant. The body weight (**A**) and serum ALT level (**B**) of the transgenic mice were measured immediately before treatment and once a week or month after treatment of normal diets (control), unformulated or phytosomal curcumin diets for 6 consecutive months. Data represent the mean with standard error of the mean (SEM) error bar.

To assess the effect of phytosomal curcumin on physiological function of mice, the body weight of transgenic mice expressing both HBx and pre-S2 mutant was measured immediately before treatment and once a week after treatment of normal diets, unformulated or phytosomal curcumin diets. As shown in Fig. 2A, the body weight of mice treated with either unformulated or phytosomal curcumin diets was not affected, showing a similar gradual increase with the normal diets-treated control mice during the treatment period. Moreover, the liver function of the transgenic mice was evaluated by measuring serum ALT. As shown in Fig. 2B, serum ALT level in mice treated with either unformulated or phytosomal curcumin diets was moderately lower than that in the control mice, although not in a statistically significant manner, suggesting that either unformulated or phytosomal curcumin treatment would not cause liver injury in mice.

### Phytosomal curcumin exhibited chemopreventive effects on HCC development in transgenic mice expressing both HBx and pre-S2 mutant

**Figure 3:**
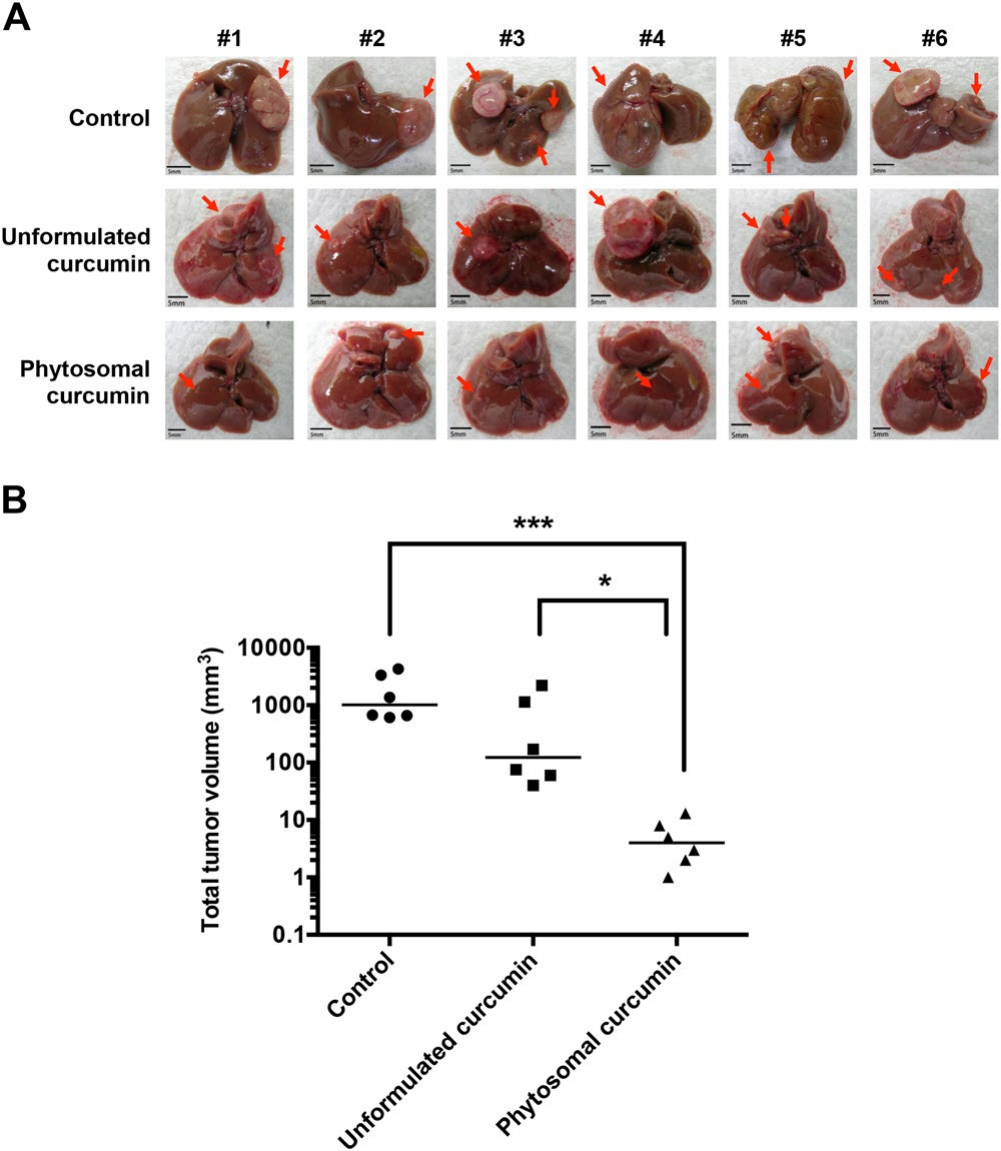
Phytosomal curcumin inhibited HCC formation and reduced the total tumor volume in liver of transgenic mice expressing both HBx and pre-S2 mutant. (**A**) The formation of tumor in the transgenic mice liver was examined at the end of treatment of normal diets (control), unformulated or phytosomal curcumin diets. Tumors were encircled by red dashed lines and indicated by red arrows. Scale bar, 5 mm. (**B**) The total tumor volume in the transgenic mice liver from each treatment group was determined. The y axis is on the log scale. Horizontal lines represent the median values of the distribution. A statistical significance of the difference between the control mice and phytosomal curcumin diets-treated mice rather than the unformulated curcumin diets-treated mice was shown. A statistical significance of the difference between the phytosomal curcumin diets- and unformulated curcumin diets-treated mice was also observed. *P < 0.05, ***P < 0.001.

To evaluate the effectiveness of phytosomal curcumin in preventing the development of HBV-related HCC, the transgenic mice expressing both HBx and pre-S2 mutant were administered with normal diets or diets containing unformulated or phytosomal curcumin for 6 months. After the treatment, mice were sacrificed to examine the formation of tumor in liver. As shown in Fig. 3A, HCC formation was slightly suppressed in the unformulated curcumin diets-treated mice but apparently inhibited in the phytosomal curcumin diets-treated mice compared with the normal diets-treated control mice. The total tumor volume (median, range) was significantly reduced in the phytosomal curcumin diets-treated mice (4 mm^3^, 1 to 13 mm^3^) rather than the unformulated curcumin diets-treated mice (123 mm^3^, 40 to 2200 mm^3^) compared with the control mice (1018 mm^3^, 611 to 4280 mm^3^) (Fig. 3B). A significant difference in total tumor volume was also observed between the phytosomal curcumin diets- and unformulated curcumin diets-treated mice (Fig. 3B).

**Figure 4:**
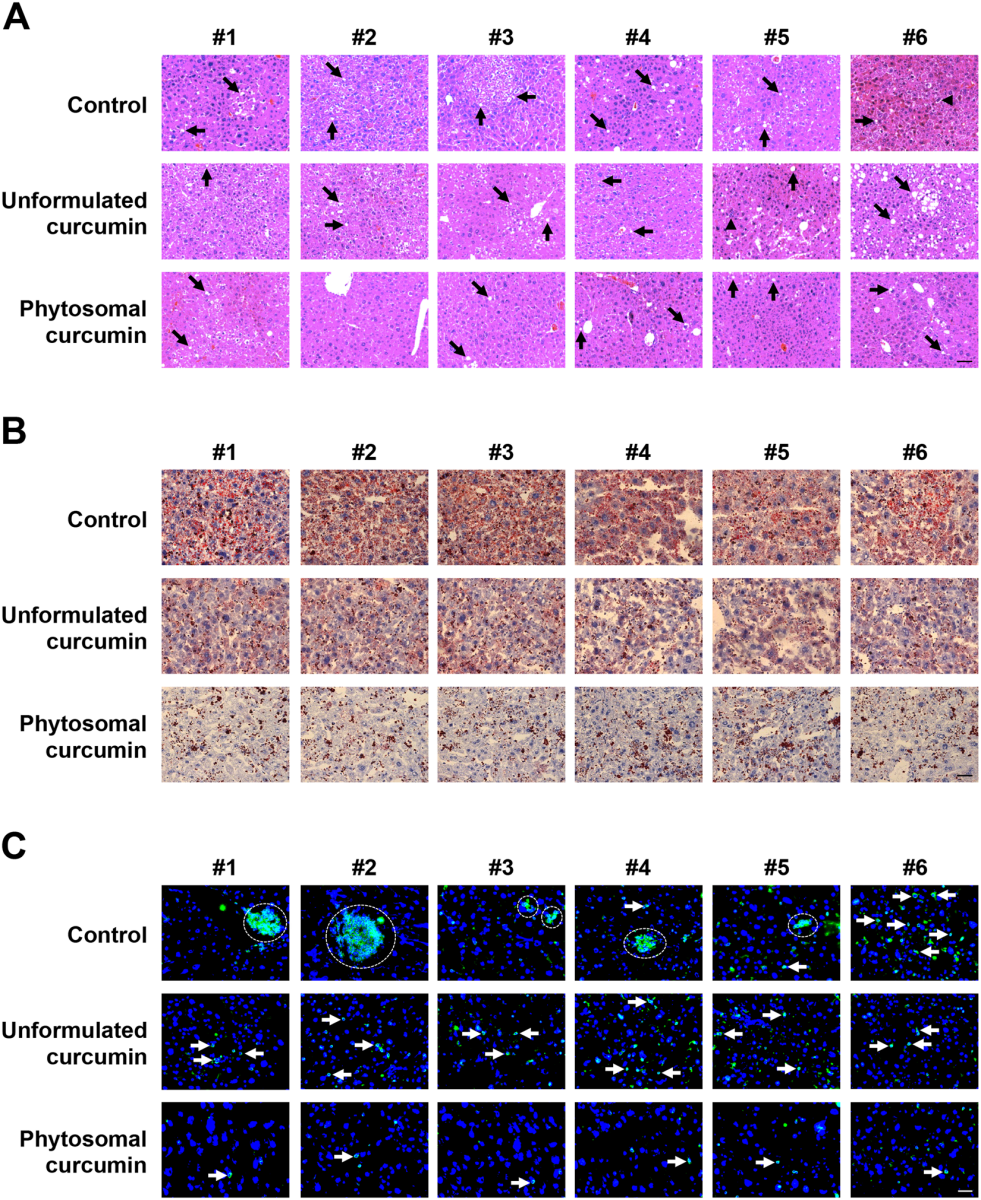
Phytosomal curcumin improved histopathology and decreased lipid accumulation and leukocyte infiltration in liver of transgenic mice expressing both HBx and pre-S2 mutant. (**A**) The histopathology of the transgenic mice liver was examined at the end of treatment of normal diets (control), unformulated or phytosomal curcumin diets by H&E staining. Black arrows indicate the steatosis (small lipid droplets) and black arrowheads indicate the necroinflammation (inflammatory cell clusters). Shown were representative results of each mouse. Original magnification, ×20. Scale bar, 100 μm. (**B**) The intracellular lipid deposit (red in color) in liver of each treatment group of mice was evaluated by Oil Red O staining. (**C**) The expression of CD45 (green in color) in liver tissues of each treatment group of mice was detected by fluorescent IHC staining. Nuclei were stained with DAPI (blue in color). White arrows and white dashed circles indicate the single and clustered CD45-positive cells, respectively. Shown were representative results of each mouse. Original magnification, ×40. Scale bar, 50 μm.

Furthermore, the histopathology of mice liver was examined by hematoxylin and eosin (H&E) staining. As shown in Fig. 4A, the phytosomal curcumin diets-treated mice revealed improved histopathology of liver that showed only mild steatosis but not necroinflammation compared with the control mice and unformulated curcumin diets-treated mice. The level of steatosis in liver tissues was further evaluated by Oil Red O staining for the accumulation of intracellular lipid droplets. As shown in Figs 4B and S1A, the control mice displayed an extensive accumulation of lipids in liver tissues. Compared with the control mice, the level of lipid accumulation in liver tissues was moderately decreased in the unformulated curcumin diets-treated mice but considerably reduced in the phytosomal curcumin diets-treated mice, suggesting that the phytosomal curcumin diets-treated mice displayed milder steatosis than the unformulated curcumin diets-treated and control mice. Moreover, the infiltration of inflammatory cells in liver tissues was examined by fluorescent immunohistochemistry (IHC) staining of the common leukocyte antigen CD45. As shown in Figs 4C and S1B, the number of CD45-positive cells in liver tissues was considerably lower in the phytosomal curcumin diets-treated mice than the unformulated curcumin diets-treated and control mice, indicating that phytosomal curcumin had a greater anti-inflammatory effect on liver during tumorigenesis.

### Phytosomal curcumin displayed cytotoxic effects on the growth of HCC cells

**Figure 5:**
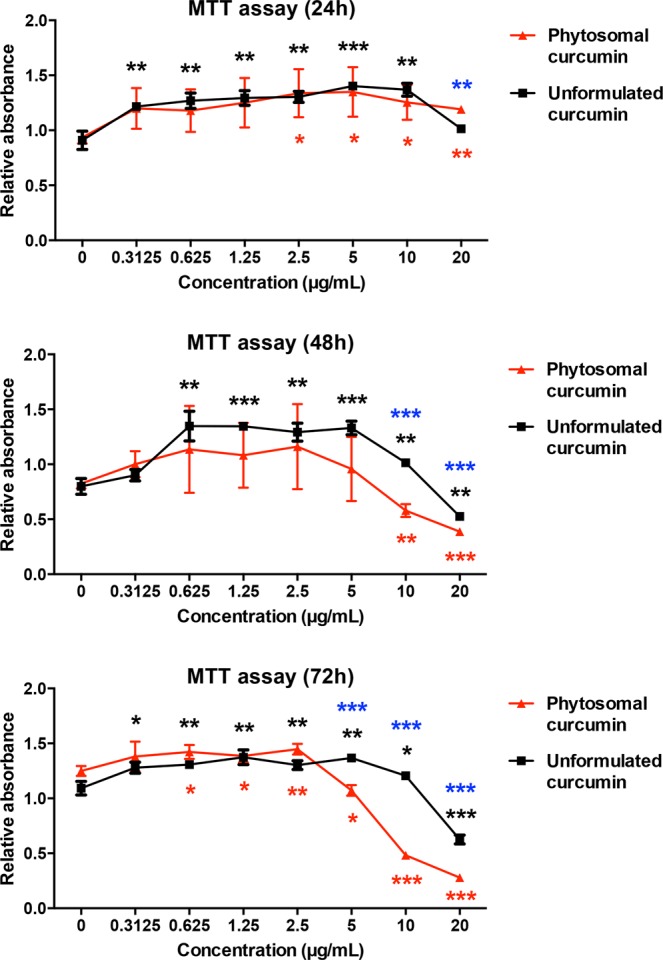
Phytosomal curcumin suppressed the growth of HCC cells. HuH-7 cells were either left untreated or treated with unformulated or phytosomal curcumin with the indicated working concentrations for 24, 48, and 72 hours. At the indicated time points, the level of cell growth in each treatment was determined by MTT assay for measuring the absorbance values at 450 nm. Data represent the mean relative to the untreated cells from three independent experiments. Error bars indicate SD. Black and red asterisks denote the statistically significant differences between the untreated cells and either unformulated or phytosomal curcumin-treated cells, respectively. The statistically significant differences between the unformulated and phytosomal curcumin-treated cells were further indicated by blue asterisks. *P < 0.05, **P < 0.01, ***P < 0.001.

To ascertain the effect of phytosomal curcumin on the growth of HCC cells, HuH-7 cells were either left untreated or treated with unformulated or phytosomal curcumin with the indicated concentrations for 24, 48, and 72 hours. At the indicated time points, cell viability assay was performed to determine the level of cell growth in each treatment. As shown in Fig. 5, compared with the untreated control, either unformulated or phytosomal curcumin dose-dependently suppressed the growth of HuH-7 cells at 48 and 72 rather than 24 hours after the treatment. Remarkably, the phytosomal curcumin showed a stronger cytotoxic activity against HuH-7 cell growth than the unformulated curcumin.

### Phytosomal curcumin exerted inhibitory effects on both pro-inflammatory and oncogenic signal activation

**Figure 6:**
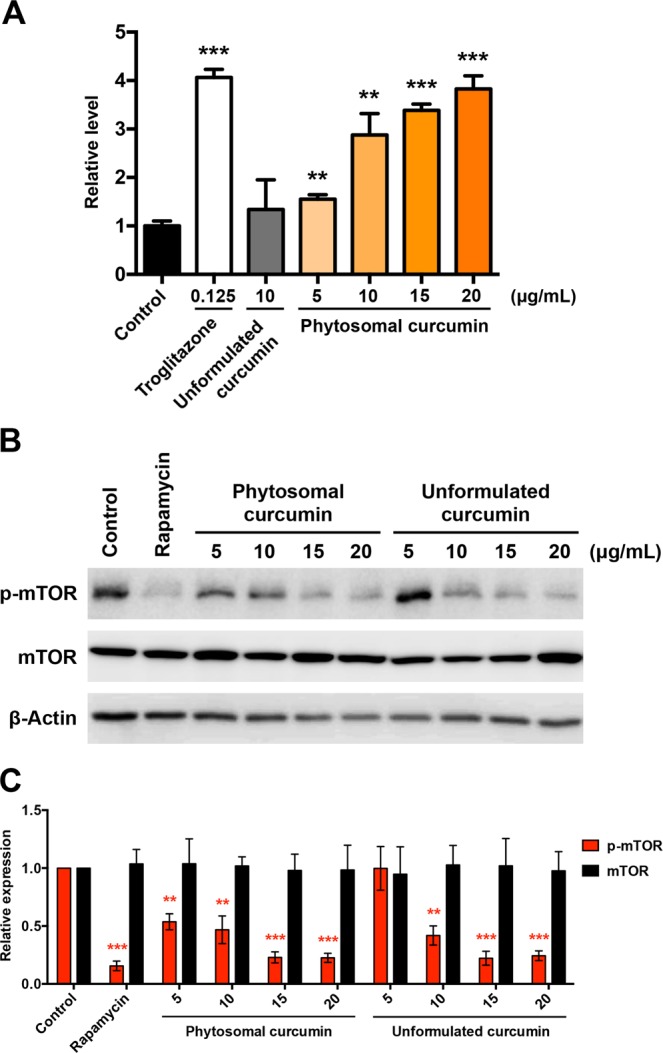
Phytosomal curcumin enhanced PPARγ activity and suppressed mTOR activation in HCC cells. (**A**) HuH-7 cells were transfected with a GAL4-driven reporter plasmid and a plasmid encoding the PPARγ LBD fused with the GAL4 DNA-binding domain. After transfection, the cells were either left untreated (control) or treated with the indicated working concentrations of troglitazone, unformulated curcumin, or phytosomal curcumin. The activity of PPARγ in the cells was measured by luciferase reporter assay. Data represent the mean relative to the control cells. Error bars indicate SD. Black asterisks denote the statistically significant differences between the treated and control cells. **P < 0.01, ***P < 0.001. (**B**) HuH-7 cells were either left untreated (control) or treated with a working concentration of 200 nM of rapamycin or with the indicated working concentrations of unformulated or phytosomal curcumin. After treatment, the expression of phosphorylated (p) activated form of mTOR was detected by Western blot analysis. Shown were representative results from three independent experiments. (**C**) Quantitative and statistical analysis of the Western blotting data. Data in each experiment were presented as mean values relative to the control cells. Error bars indicate SD. Red asterisks denote the statistically significant differences between the treated and control cells. **P < 0.01, ***P < 0.001.

To elucidate the mechanisms by which phytosomal curcumin exerted its chemopreventive effects on HCC, the effect of phytosomal curcumin on the activity of PPARγ, NF-κB, and mTOR was examined. To assess the effect of phytosomal curcumin on PPARγ activity, HuH-7 cells were transfected with a GAL4-driven reporter plasmid and a plasmid encoding the PPARγ LBD fused with the GAL4 DNA-binding domain. As shown in Fig. 6A, treatment of these cells with phytosomal curcumin significantly induced a higher level of transcriptional activity of PPARγ than that induced by equal concentration of unformulated curcumin. Moreover, phytosomal curcumin showed a dose-dependent activation of PPARγ by approximately 4-fold activation with a concentration of 20 μg/mL comparable to that with a concentration of 0.125 μg/mL by troglitazone that served as a positive control for PPARγ activation. In addition, the effect of phytosomal curcumin on mTOR activity was assayed in HuH-7 cells by Western blot analysis. As shown in Fig. 6B,C, treatment of either phytosomal or unformulated curcumin with a concentration of 20 μg/mL decreased the level of phosphorylated activated form of mTOR to a similar extent to that by a concentration of 200 nM of rapamycin, an mTOR inhibitor.

**Figure 7:**
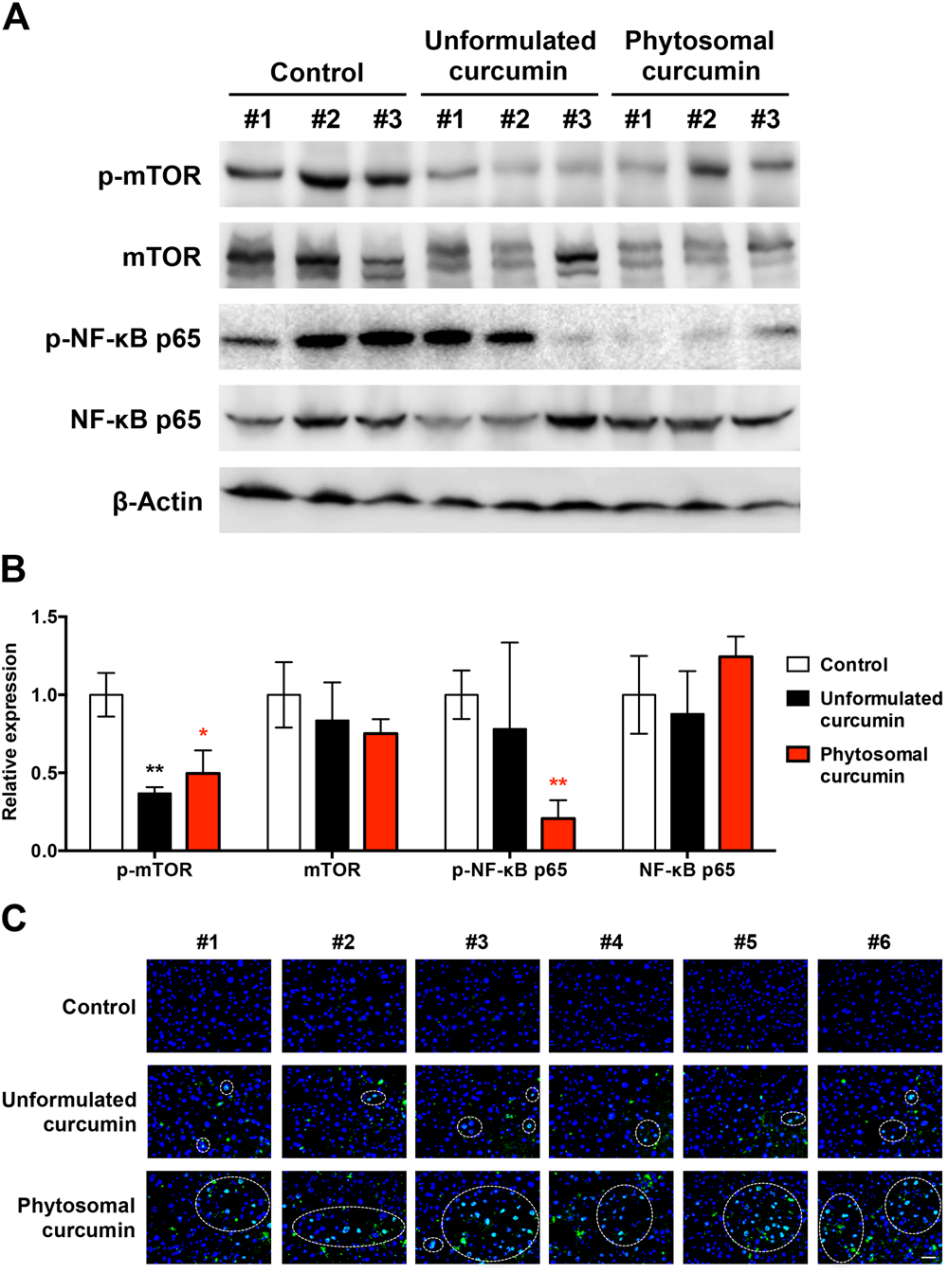
Phytosomal curcumin activated PPARγ but inhibited both NF-κB and mTOR activity in liver of transgenic mice expressing both HBx and pre-S2 mutant. (**A**) Liver tissues were isolated from the transgenic mice at the end of treatment of normal diets (control), unformulated or phytosomal curcumin diets. The expression of phosphorylated (p) activated form of NF-κB p65 and mTOR was detected by Western blot analysis. Three livers were used in each treatment group. (**B**) Quantitative and statistical analysis of the Western blotting data. Data in each treatment group of mice were presented as mean values relative to the control mice. Error bars indicate SD. Black and red asterisks denote the statistically significant differences between the control mice and either unformulated or phytosomal curcumin diets-treated mice, respectively. *P < 0.05, **P < 0.01. (**C**) Detection of nuclear PPARγ expression in liver tissues of each treatment group of mice by fluorescent IHC staining. Localization of PPARγ (green in color) in the nucleus (blue in color) appeared cyan and was highlighted by white dashed circles. Shown were representative results of each mouse. Original magnification, ×40. Scale bar, 50 μm.

To further ascertain the effect of phytosomal curcumin on NF-κB and mTOR activity in the transgenic mice expressing both HBx and pre-S2 mutant, liver tissues were isolated at the end of the treatment for Western blot analysis. As shown in Fig. 7A,B, the levels of phosphorylated activated form of NF-κB p65 and mTOR in mice liver were consistently suppressed in the phytosomal curcumin diets-treated mice compared with the normal diets- and unformulated curcumin diets-treated mice, although the level of phosphorylated mTOR was also reduced in the unformulated curcumin diets-treated mice. Next, we assessed the activity of PPARγ in mice liver by performing fluorescent IHC staining to detect the level of nuclear expression of PPARγ in liver tissues. Previous research has shown that upon activation PPARγ can translocate into the nucleus where it transcriptionally regulates the expression of its target genes^44^. As shown in Figs 7C and S2, the number of cells with nuclear PPARγ expression in liver tissues was apparently increased in the phytosomal curcumin diets-treated mice compared with the normal diets- and unformulated curcumin diets-treated mice, suggesting that phytosomal curcumin diets-treated mice exhibited higher level of PPARγ activation in liver than the normal diets- and unformulated curcumin diets-treated mice.

## Discussion

**Figure 8:**
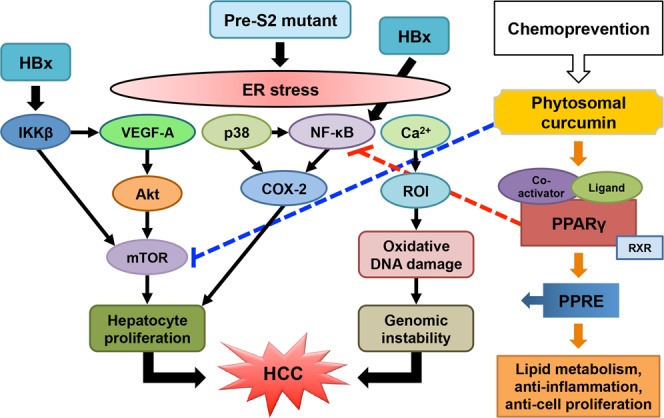
Schematic model for the chemopreventive effect of phytosomal curcumin on HBV-related HCC. In chronic HBV infection, two HBV oncoproteins, HBx and pre-S2 mutant, play key roles in the progression of HCC through either the induction of ER stress-induced oxidative DNA damage to cause genomic instability or the activation of NF-κB and mTOR signal pathways to promote hepatocyte proliferation. Phytosomal curcumin may exert its chemopreventive effects on HBV-related HCC through three mechanisms: one involving the activation of PPARγ activity to upregulate the expression of genes involved in lipid metabolism, anti-inflammation, and anti-cell proliferation (as indicated by sequential orange arrows), another involving the inhibition of NF-κB activation though PPARγ to repress the expression of pro-inflammatory cytokines (as indicated by red dashed lines), and the other involving the suppression of mTOR activation to block hepatocyte proliferation (as indicated by blue dashed lines). Abbreviations: HBV, hepatitis B virus; HBx, hepatitis B X protein; HCC, hepatocellular carcinoma; ER, endoplasmic reticulum; NF-κB, nuclear factor-κB; mTOR, mammalian target of rapamycin; PPARγ, peroxisome proliferator-activated reporter γ; RXR, retinoid X receptor; PPRE, peroxisome proliferator response element; VEGF-A, vascular endothelial growth factor-A; COX-2, cyclooxygenase-2; IKKβ, IκB kinase β; Ca^2+^, ionized calcium; ROI, reactive oxygen intermediates.

Although considerable progress has been made in the prevention and therapy of HBV-related HCC, the efficacy of current treatments is still limited. The major obstacles are the persistence of HBV surface antigen expression from the integrated HBV DNA as well as the emergence of side effects during the long-term treatment^11,14^. Indeed, the presence of pre-S2 mutant exhibits a high resistance to anti-viral therapies and carries a high risk of HCC development in chronic HBV carriers^12^. Therefore, natural products with active ingredients and without side effects would serve as ideal chemopreventive agents against HCC in patients with chronic HBV infection. In this study, we for the first time evaluated the effectiveness of phytosomal formulation of curcumin in the treatment of HBV-related HCC and demonstrated that phytosomal curcumin exhibited a remarkable chemopreventive effect on HBV-related HCC, at least in part, through the activation of PPARγ and the inhibition of NF-κB and mTOR activities (Fig. 8).

In this study, we used the transgenic mice specifically expressing double HBV oncoproteins (HBx and pre-S2 mutant) in liver as a model of HBV-related hepatocarcinogenesis to evaluate the chemopreventive effect of phytosomal curcumin. HBx and pre-S2 mutant have been well demonstrated as the two most important HBV oncoproteins that play key roles in the progression of HCC in chronic HBV infection^15,16^. As summarized in Fig. 8, pre-S2 mutant can induce ER stress signals, which cause oxidative stress and DNA damage, leading to genomic instability^45,46^. Through the induction of ER stress, pre-S2 mutant can also activate two signal pathways to promote hepatocyte proliferation, one involving NF-κB and p38 to upregulate cyclooxygenase-2 (COX-2) and another involving vascular endothelial growth factor-A (VEGF-A) and Akt to activate mTOR^47,48^. In addition, HBx has been shown to activate NF-κB signal pathways through several mechanisms^49–51^. HBx can also upregulate mTOR signaling through IκB kinase β (IKKβ) to increase cell proliferation and VEGF production^52^. Activation of mTOR has been shown to play essential roles in the development of HBV-related HCC^53^, and is a major molecular event throughout the liver tumorigenesis in the transgenic mice harboring both HBx and pre-S2 mutant^23^. In addition, the liver tissues of transgenic mice expressing either or both of pre-S2 mutant and HBx have been shown to display several dysplastic changes and represent a preneoplastic lesion for HCC development^23,42,43,54–56^. Therefore, the data from our mouse model may provide supportive evidence for the chemopreventive effect of phytosomal curcumin on HBV-related HCC development.

Several formulation strategies have been employed to overcome the bioavailability limitations of curcumin^37^. Among them, phytosomes have a better stability than many other formulations due to the covalent bonds formed between phospholipid and curcumin in phytosomes^39^. As a result, phytosomal curcumin has been shown to display enhanced bioavailability and improved pharmacokinetics compared with unformulated curcumin^39,40^. Consistent with the advantages and properties of phytosomal curcumin, in this study, we demonstrated that phytosomal curcumin exhibited significantly greater effects on suppression of HCC formation, improvement of liver histopathology (steatosis and necroinflammation), and reduction of total tumor volume than unformulated curcumin in the transgenic mice expressing both HBx and pre-S2 mutant. Moreover, compared with unformulated curcumin, phytosomal curcumin exerted considerably stronger effects on activation of anti-inflammatory PPARγ as well as inhibition of pro-inflammatory NF-κB. Also, phytosomal curcumin could suppress oncogenic mTOR activation to a similar extent to unformulated curcumin. As summarized in Fig. 8, we therefore proposed that phytosomal curcumin may exert its chemopreventive effects on HBV-related HCC, at least in part, through the following three mechanisms: First, phytosomal curcumin may act as an agonist to activate PPARγ. Upon activation, PPARγ forms heterodimers with the retinoid X receptor (RXR), recruits co-activators, and binds to the peroxisome proliferator response element (PPRE), leading to the upregulation of genes involved in lipid metabolism, anti-inflammation, and anti-cell proliferation^57^. Second, the activated PPARγ by curcumin may additionally inhibit NF-κB activation through several mechanisms, resulting in the repression of NF-κB-mediated transcription of pro-inflammatory cytokines^58–60^. Last but not the least, phytosomal curcumin may also suppress the activation of mTOR-mediated oncogenic signal pathways^29,61^.

Furthermore, in this study, the expression of pre-S2 mutant and HBx in liver tissues of each treatment group of mice was also examined by fluorescent IHC staining. As shown in Figs S3 and S4, both pre-S2 mutant and HBx were consistently expressed in liver tissues at similar levels between normal diets-, unformulated curcumin diets-, and phytosomal curcumin diets-treated mice, suggesting that either unformulated or phytosomal curcumin exerted its effect on PPARγ, NF-κB, and mTOR activities not through targeting pre-S2 mutant and HBx directly. Therefore, it may be applicable to use the human hepatoma HuH-7 cell line as a culture system to investigate the underlying mechanisms of phytosomal curcumin in HCC chemoprevention.

In conclusion, our results suggest that the combined beneficial activities of anti-inflammation, lipid metabolism, and anti-cell proliferation may potentially contribute to the effectiveness of phytosomal curcumin on prevention of HBV-related HCC development. Phytosomal curcumin may have great promise as a chemopreventive agent against HCC for the long-term treatment of patients with chronic HBV infection.

## Methods

### Unformulated and phytosomal curcumin

The unformulated curcumin (a mixture of 95% curcuminoids, comprising curcumin, demethoxycurcumin, and bisdemethoxycurcumin) was obtained from Sigma, Louis, MO, USA. The phytosomal curcumin, Meriva^®^ (a curcumin-phosphatidylcholine complex containing 20% curcuminoids), was purchased from Indena SpA, Milan, Italy. For animal experiment, the unformulated and phytosomal curcumin were mixed with a normal chow diet (Research Diets, New Brunswick, NJ, USA) to prepare the unformulated and phytosomal curcumin diets, respectively, each containing 1 mg curcuminoids/g.

### Curcumin administration in mice

For curcumin treatment, the mice were randomized into one of three treatment groups (6 mice/group): the normal diets (control group), the unformulated curcumin diets (150 mg curcuminoids/kg body weight/day), and the phytosomal curcumin diets (150 mg curcuminoids/kg body weight/day) treatment groups. Each mouse was weighted and given a maintenance diet for 7 days (total dose, 1050 mg curcuminoids/kg body weight) every week during 6 months of treatment. All animal experiments were performed in male mice under the approval of the institutional animal care and use committee of the National Health Research Institutes, Tainan, Taiwan (Approval No: NHRI-IACUC-104114 and NHRI-IACUC-106152-A). All research was performed in accordance with relevant guidelines and regulations.

### Body weight and ALT measurement

The body weight of each mouse was recorded immediately before treatment and once a week after treatment for 6 consecutive months. For ALT measurement, serum samples were obtained from mice immediately before treatment and once a month after treatment until sacrifice. ALT level was measured by FUJIFILM DRI-CHEM slides using FUJIFILM DRI-CHEM 3500 machine (FUJIFILM Corporation, Tokyo, Japan).

### Liver histopathology and tumor volume analysis

At sacrifice, the isolated mice livers were imaged and examined for tumor formation. To determine tumor volume, the greatest longitudinal diameter (length) and the greatest transverse diameter (width) were measured by caliper. The tumor volume was calculated by the formula tumor volume = 1/2 (length × width^2^). To examine liver histopathology, the formalin-fixed and paraffin-embedded liver tissues were sectioned for H&E staining. To evaluate lipid deposition in liver, the frozen liver tissues were sectioned for Oil Red O staining (Muto Pure Chemicals, Tokyo, Japan) as described^54^. For quantification, five independent microscopic fields (original magnification, ×40) with the most abundant lipid droplets in liver tissues of each mouse were selected. The percent area of lipid droplets in the five selected fields of each mouse was quantified by the ImageJ software (http://rsb.info.nih.gov/ij) and further calculated as the percent area of lipid droplets per field for statistical analysis.

### Plasmid, cell line, and transient transfection

The pBIND-PPARγ-LBD plasmid was constructed by cloning the PPARγ ligand-binding domain (LBD) (amino acids 172-476) PCR products into the pBIND vector (Promega, Madison, WI, USA), which fused with the GAL4 DNA-binding domain. The GAL4-pGL4-luc reporter plasmid was constructed by inserting the 5 GAL4 response elements into the promoter region of the pGL4.17 reporter plasmid (Promega). The pRL-TK Renilla luciferase vector was purchased from Promega. The human hepatoma HuH-7 cell line was obtained from the Health Science Research Resources Bank (JCRB0403; Osaka, Japan). All transfections were performed with the Lipofectamine 2000 reagent (Invitrogen, Carlsbad, CA, USA) according to the manufacturer’s instructions.

### Cell viability assay

At the indicated time points, cells were subjected to MTT (3-[4, 5-dimethylthiazol-2-yl]-2, 5 diphenyl tetrazolium bromide) colorimetric assay (Sigma) according to the manufacturer’s instructions. Absorbance values at 450 nm were measured to determine the level of cell viability. The experiments were performed in triplicate three times independently.

### Luciferase reporter assay

HuH-7 cells were transfected with pBIND-PPARγ-LBD plasmid, GAL4-pGL4-luc reporter plasmid, and pRL-TK plasmid. Twenty-four hours after transfection, the cells were either left untreated or treated with troglitazone (Sigma), unformulated curcumin, or phytosomal curcumin with the indicated concentrations for another 24 hours. The luciferase-expressed cells were then assayed by the Dual-Luciferase reporter assay system (Promega) according to the manufacturer’s instructions. Renilla luciferase activity was measured for normalization. The experiments were performed in triplicate three times independently.

### Western blot analysis

HuH-7 cells were either left untreated or treated with rapamycin (Sigma), unformulated curcumin, or phytosomal curcumin with the indicated concentrations for 24 hours. Western blot analysis was performed as described^62^. Briefly, total proteins were extracted with lysis buffer containing protease and phosphatase inhibitor cocktail (Roche Diagnostics, Mannheim, Germany) from cells or mice liver tissues after treatment. Equal amounts of proteins for each sample were resolved on sodium dodecyl sulfate- polyacrylamide gels and transferred to polyvinylidene difluoride membranes. Membranes were incubated with primary antibodies, followed by secondary antibodies, and then developed by an enhanced chemiluminescence system (Amersham Pharmacia Biotech, Amersham, UK). The primary antibodies used in this study were anti-mTOR (2972), anti-NF-κB p65 (8242), and anti-p-NF-κB p65 (Ser536) (3033) from Cell Signaling Technology (Danvers, MA, USA), anti-p-mTOR (Ser2448) (ab1093) from Abcam (Cambridge, UK), and anti-β-Actin (MAB1501) from Millipore (Billerica, MA, USA). β-Actin was used as the internal control. Full blots were shown in Fig. S5.

### Fluorescent IHC staining

Fluorescent IHC staining was performed as described^23^. Briefly, the frozen liver tissues from each treatment group of mice were sectioned and incubated with the primary antibodies anti-PPARγ (2435; Cell Signaling Technology), anti-HBsAg (1811; ViroStat, Portland, ME, USA), anti-HBx (ab39716; Abcam), or anti-CD45 (ab10558; Abcam), followed by the secondary antibody Alexa Fluor 488-conjugated goat anti-rabbit IgG (ab150077; Abcam). Anti-HBsAg was used to detect pre-S2 mutant. DAPI (4’, 6-diamidino-2-phenylindole; Invitrogen) was used to stain the nuclei. For quantification, five independent microscopic fields (original magnification, ×40) with the most abundant nuclear PPARγ-, CD45-, pre-S2 mutant-, or HBx-positive cells in liver tissues of each mouse were selected. The total number of nuclear PPARγ-, CD45-, pre-S2 mutant-, or HBx-positive cells in the five selected fields of each mouse was counted manually and further calculated as the number of nuclear PPARγ-, CD45-, pre-S2 mutant-, or HBx-positive cells per field for statistical analysis.

### Statistical analysis

The significance of the difference of body weight, ALT level, total tumor volume, and the percent area of lipid droplets, as well as nuclear PPARγ-, CD45-, pre-S2 mutant-, and HBx-positive cell number between the normal diets, unformulated curcumin diets, and phytosomal curcumin diets treatment groups of mice was determined by Kruskal-Wallis one-way ANOVA followed by Dunn’s multiple comparisons test. The significance of the difference of signaling molecule expression between different treatment groups of mice livers was determined by unpaired *t*-test. The significance of the difference of cell viability, PPARγ activity, and signaling molecule expression between the untreated control cells and the cells treated with troglitazone, rapamycin, unformulated curcumin, or phytosomal curcumin was determined by unpaired *t*-test. A *P* value < 0.05 was considered significant (*P < 0.05, **P < 0.01, ***P < 0.001).

## Supplementary information

Supplementary information

## Data Availability

All data generated or analyzed during this study are included in this published article and its Supplementary Information files.
