# Supplementary material for: Chemopreventive Effect of Phytosomal Curcumin on Hepatitis B Virus-Related Hepatocellular Carcinoma in A Transgenic Mouse Model
Source: Sci Rep. 2019 Jul 17;9:10338. doi: 10.1038/s41598-019-46891-5 (PMC6637187; doi:10.1038/s41598-019-46891-5)
Supplement: Supplementary file 1 — Supplementary information [file 41598_2019_46891_MOESM1_ESM.pdf]

**Chemopreventive Effect of Phytosomal Curcumin on Hepatitis B Virus-Related Hepatocellular Carcinoma in A Transgenic Mouse Model**

Chiao-Fang Teng,[1,2] Chun-Hui Yu,[3] Hong-Yi Chang,[3] Wen-Chuan Hsieh,[4] Tzu-Hua Wu,[2] Jia-Hui Lin,[1] Han-Chieh Wu,[4] Long-Bin Jeng,[2] Ih-Jen Su[3,4,5]

[1]Graduate Institute of Biomedical Sciences, China Medical University, Taichung, Taiwan
[2]Organ Transplantation Center, China Medical University Hospital, Taichung, Taiwan
[3]Department of Biotechnology, Southern Taiwan University of Science and Technology, Tainan, Taiwan
[4]National Institute of Infectious Diseases and Vaccinology, National Health Research Institutes, Tainan, Taiwan
[5]Department of Pathology, National Cheng Kung University Hospital, Tainan, Taiwan

**Correspondence**
Ih-Jen Su, M.D., Ph.D., Department of Biotechnology, Southern Taiwan University of Science and Technology, No. 1 Nantai Street, Yongkang District, Tainan City 710, Taiwan (R.O.C.). Tel: 886-6-700-0123; Fax: 886-6-208-3466; E-mail: suihjen0704@stust.edu.tw

**Short Title**
Chemoprevention of HBV-Related HCC by Phytosomal Curcumin

**Number of figures and tables**
8 figures and no tables

# Figure S1

**A**

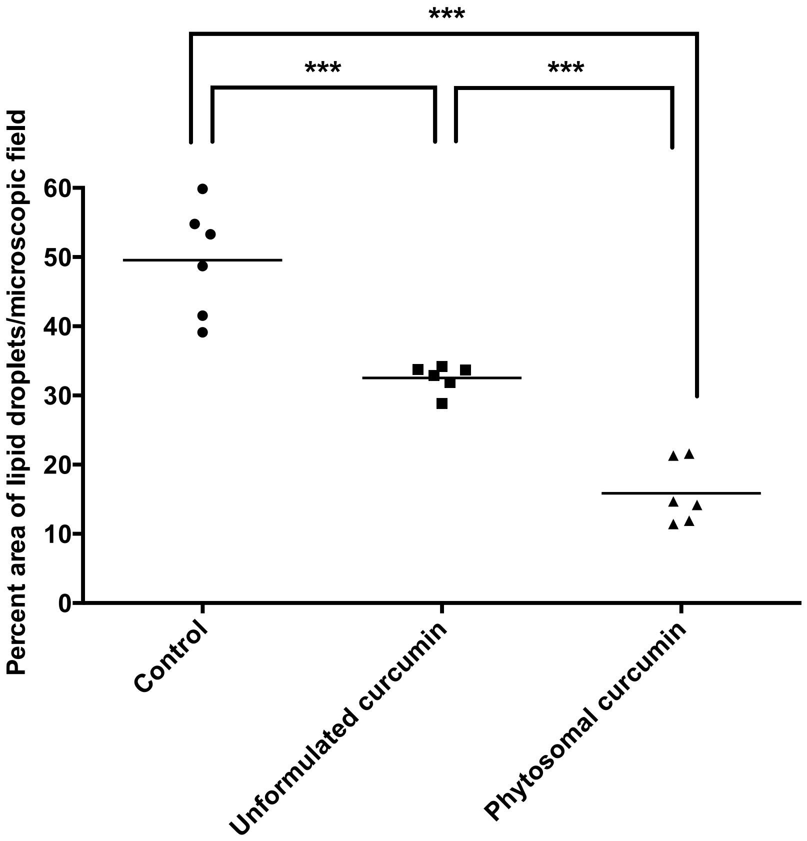
***
***
***
Percent area of lipid droplets/microscopic field
60
50
40
30
20
10
0
Control
Unformulated curcumin
Phytosomal curcumin

**B**

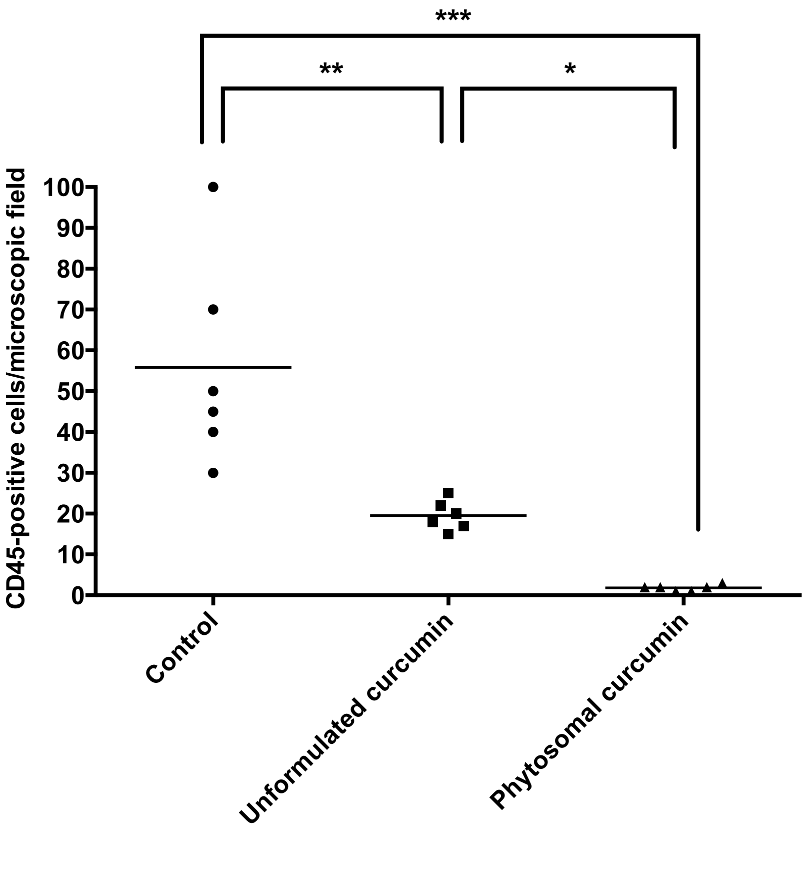
***
**
*
CD45-positive cells/microscopic field
100
90
80
70
60
50
40
30
20
10
0
Control
Unformulated curcumin
Phytosomal curcumin

# Figure S2

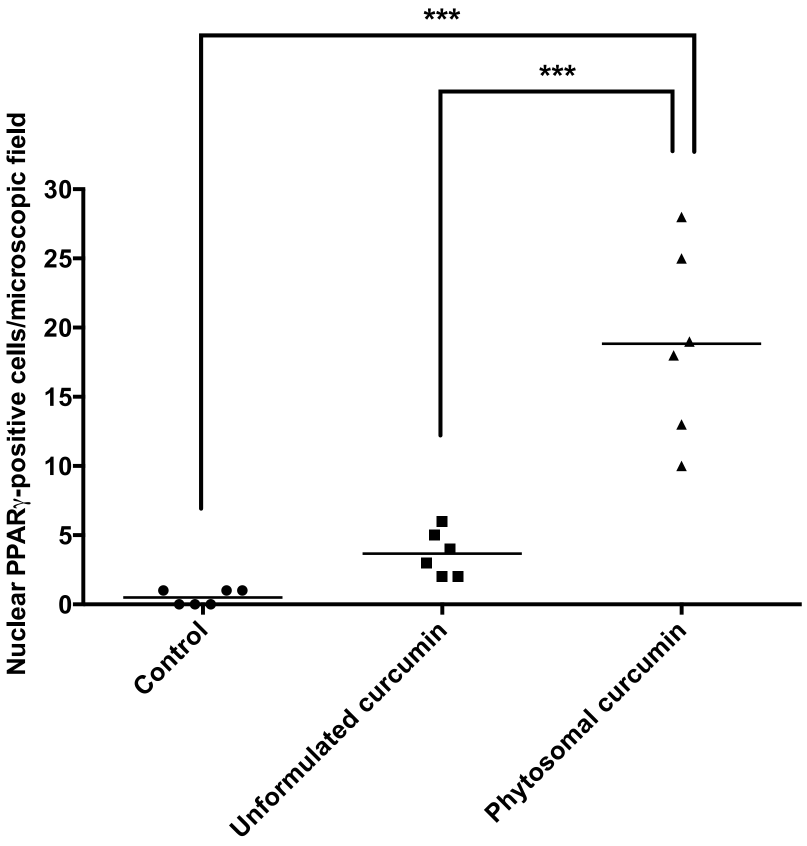
***
***
Nuclear PPARγ-positive cells/microscopic field
30
25
20
15
10
5
0
Control
Unformulated curcumin
Phytosomal curcumin

# Figure S3

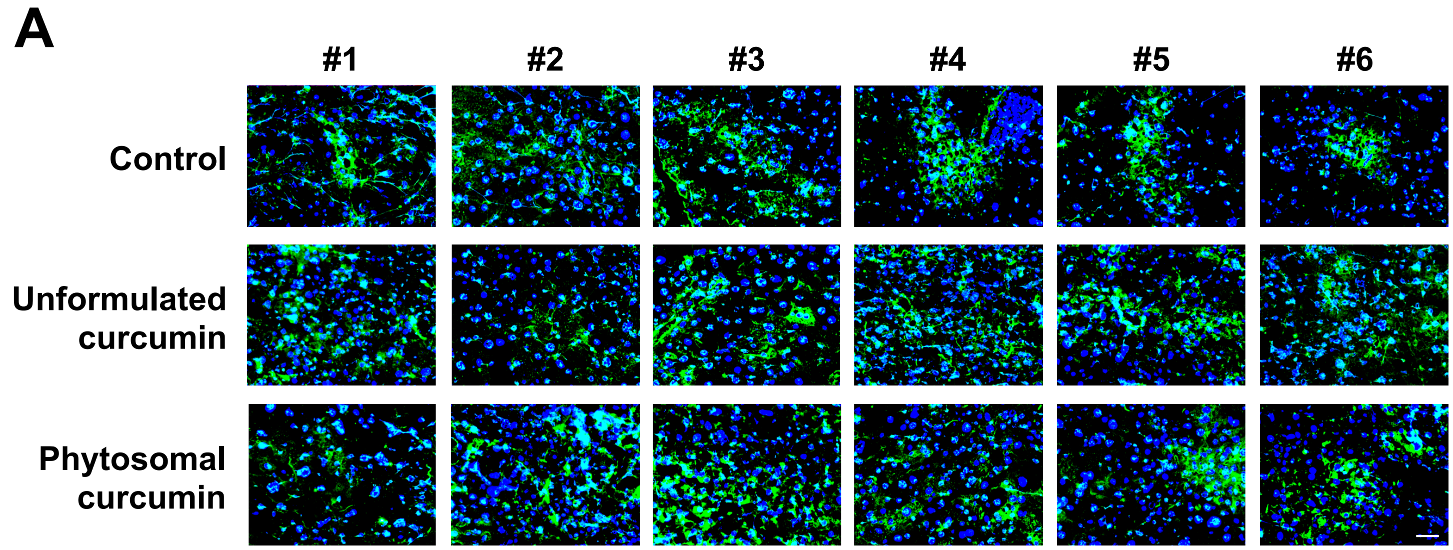
A
#1
#2
#3
#4
#5
#6
Control
Unformulated curcumin
Phytosomal curcumin

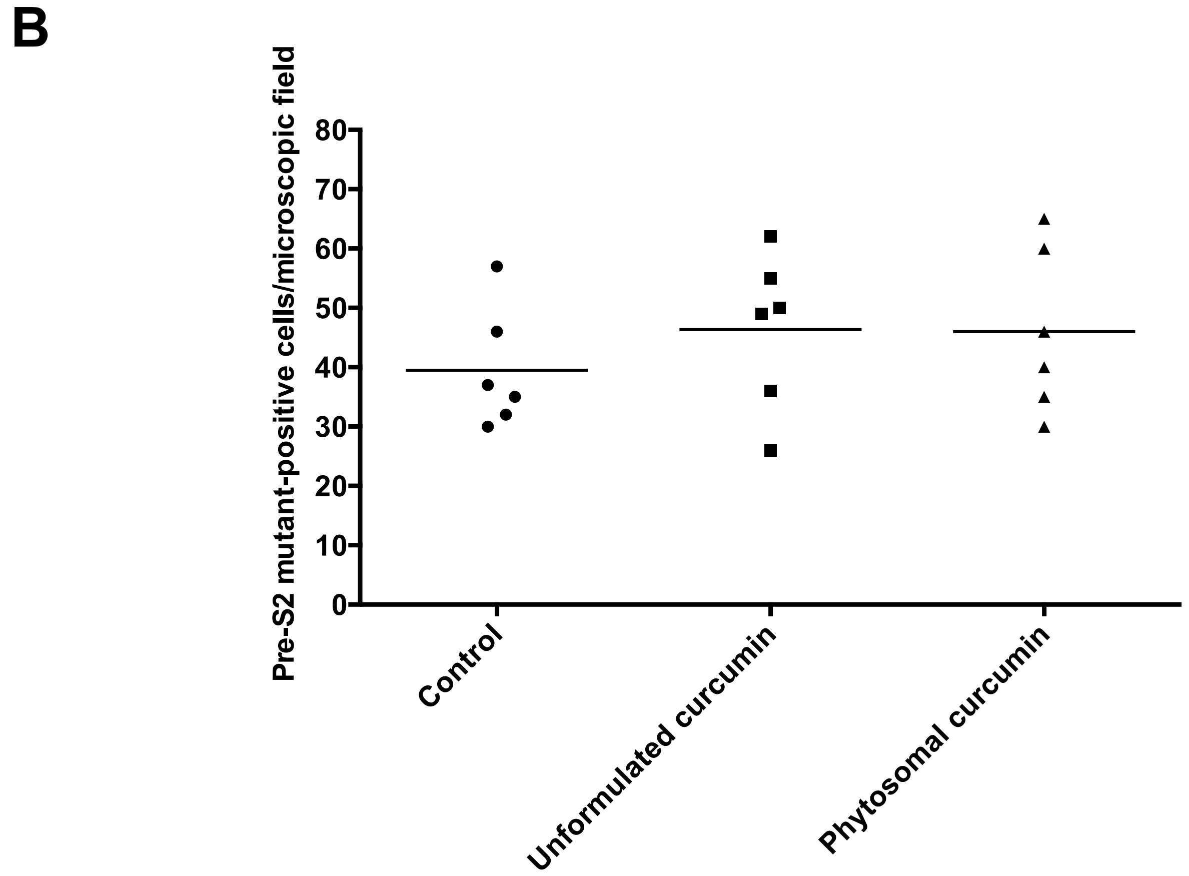
B
Pre-S2 mutant-positive cells/microscopic field
80
70
60
50
40
30
20
10
0
Control
Unformulated curcumin
Phytosomal curcumin

# Figure S4

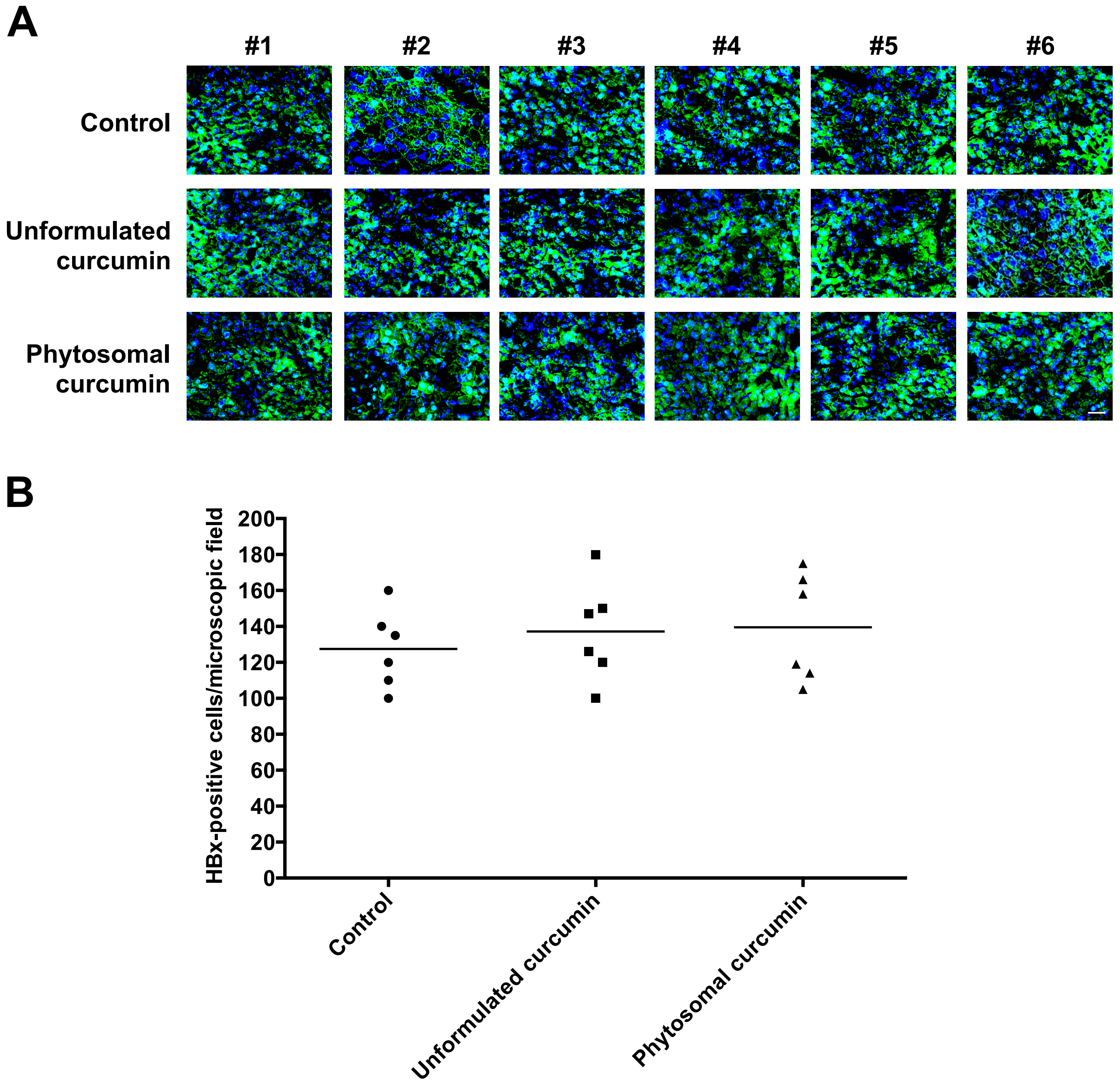
A
#1
#2
#3
#4
#5
#6
Control
Unformulated curcumin
Phytosomal curcumin
B
HBx-positive cells/microscopic field
200
180
160
140
120
100
80
60
40
20
0
Control
Unformulated curcumin
Phytosomal curcumin

# Figure S5

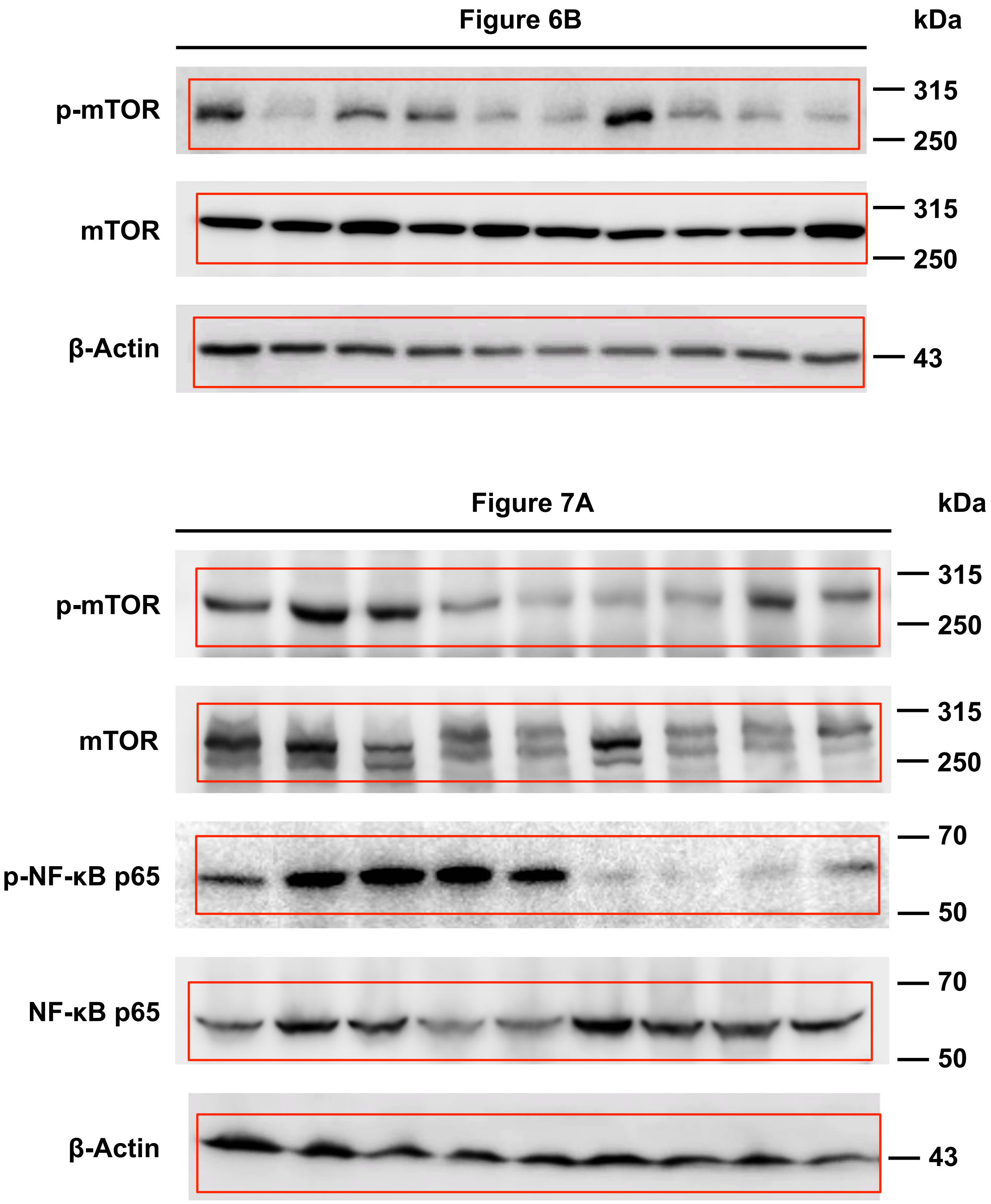

Figure 6B
kDa
p-mTOR
315
250
mTOR
315
250
β-Actin
43
Figure 7A
kDa
p-mTOR
315
250
mTOR
315
250
p-NF-κB p65
70
50
NF-κB p65
70
50
β-Actin
43

**Supplementary Figure Legends**

**Figure S1. Quantitative evaluation of decreased lipid accumulation and leukocyte infiltration in liver of transgenic mice expressing both HBx and pre-S2 mutant.** Graphs showing the percent area of lipid droplets (A) and the number of CD45-positive cells (B) per microscopic field (original magnification, ×40) in liver tissues of normal diets, unformulated curcumin diets, and phytosomal curcumin diets treatment groups of mice (n=6). The horizontal lines represented the mean values. The significance of the difference of the percent area of lipid droplets and the number of CD45-positive cells per field between different treatment groups of mice was analyzed. *$P < 0.05$, **$P < 0.01$, ***$P < 0.001$.

**Figure S2. Quantitative evaluation of PPARγ activation in liver of transgenic mice expressing both HBx and pre-S2 mutant.** Graph showing the number of nuclear PPARγ-positive cells per microscopic field (original magnification, ×40) in liver tissues of normal diets, unformulated curcumin diets, and phytosomal curcumin diets treatment groups of mice (n=6). The horizontal lines represented the mean values. The significance of the difference of the number of nuclear PPARγ-positive

cells per field between different treatment groups of mice was analyzed. ***$P <$ 0.001.

**Figure S3. Pre-S2 mutant was consistently expressed in liver of transgenic mice expressing both HBx and pre-S2 mutant.** (A) Expression of pre-S2 mutant (green in color) in liver tissues of normal diets, unformulated curcumin diets, and phytosomal curcumin diets treatment groups of mice was detected by fluorescent IHC staining. Nuclei were stained with DAPI (blue in color). Shown were representative results of each mouse. Original magnification, ×40. Scale bar, 50 μm. (B) Graph showing the number of pre-S2 mutant-positive cells per microscopic field in liver tissues of each treatment group of mice (n=6). The horizontal lines represented the mean values. No significant difference in the number of pre-S2 mutant-positive cells per field between different treatment groups of mice was observed.

**Figure S4. HBx was consistently expressed in liver of transgenic mice expressing both HBx and pre-S2 mutant.** (A) Expression of HBx (green in color) in liver tissues of normal diets, unformulated curcumin diets, and phytosomal curcumin diets

treatment groups of mice was detected by fluorescent IHC staining. Nuclei were stained with DAPI (blue in color). Shown were representative results of each mouse. Original magnification, ×40. Scale bar, 50 µm. (B) Graph showing the number of HBx-positive cells per microscopic field in liver tissues of each treatment group of mice (n=6). The horizontal lines represented the mean values. No significant difference in the number of HBx-positive cells per field between different treatment groups of mice was observed.

**Figure S5. Full immunoblots with indicated areas of selection.**
